# Taking Wishes and Feelings Seriously: The Views of People Lacking Capacity in Court of Protection Decision-Making

**DOI:** 10.1080/09649069.2014.886878

**Published:** 2014-03-05

**Authors:** Nell Munro

**Affiliations:** 1 School of Law, University of Nottingham, Nottingham, UK

**Keywords:** mental capacity, Court of Protection, dignity, best interest, autonomy

## Abstract

The Mental Capacity Act requires that where a person (P) lacks capacity to make a decision her wishes and feelings be taken into account when deciding what is in her best interests. This article considers how the Court of Protection evaluates evidence from P concerning her wishes and feelings. It finds that the Court ignores evidence regarding current wishes and fails to engage with more ambiguous evidence where P desires conflicting outcomes or may be concealing her true feelings. This is unhelpful since it makes the resulting judgment unconvincing to observers. It is legally problematic, since the Court should be following the practices of other decision-makers under the Mental Capacity Act (MCA). And it is ethically problematic since it undermines P's dignity and does not treat P as an actor whose evidence regarding her wishes and feelings has intrinsic status which the Court must make active efforts to engage with or discount rather than ignore.

## Introduction

English law requires those making substitute decisions on behalf of adults lacking capacity to undertake the challenging task of ascertaining and considering the ‘past and present wishes and feelings’ of the person in question (S, 4(6)(a) Mental Capacity Act [MCA] 2005).

In addition the decision maker should take into account the beliefs and values the person would consider if she had capacity (S. 4(6)(b), any other factors which might influence her decision (S. 4(6)(c)) and also, where practicable, consult with anyone the person might previously have identified as having awareness of her interests in the event of a loss of capacity (S. 4(7)).

In practice, the vast majority of best interests decisions made under the Mental Capacity Act are made by family members, carers and healthcare professionals without legal oversight. Empirical research has already demonstrated that people making best interests decisions under the MCA take a diverse approach to these issues with some carers attaching value to wider social norms, their own values, or placing a greater value on what they perceive to be P's wishes and feelings before she lost capacity, or the views of P's carers, or the likelihood of P recovering capacity in the future (Dunn, Clare & Holland, 2009; Williams et al., 2012). The MCA does not specify that more or less weight should be given to any of the factors in S. 4(6) and the Code of Practice does not guide decision makers to prefer any factor either. As a result provided a decision made under the Act refers to P's current wishes and feelings, even if this is given only a low priority it will still be valid in law.

The MCA is intentionally designed to empower a huge range of people to be legally authorised to make decisions on behalf of people who lack mental capacity. As such it is to be expected that diverse practices will arise. It is intended to be dynamic and responsive in its operation. This responsiveness would be inconsistent with an excessively rigid codification of the precise formula required for making best interests decisions. That is not to say the way in which the Act currently operates is ideal. There are reasonable grounds for criticism but his criticism is not the focus of this article.

We are entitled to ask for consistency indecision-making from the courts however, since this is how we ensure that like cases are treated alike. The Court of Protection has a specialist jurisdiction to hear cases relating to the operation of the Mental Capacity Act. Analysis of some recent decisions of the Court demonstrates a confusing incoherence in the way in which the Court both obtains evidence from individuals lacking capacity and evaluates it when reaching decisions upon their behalf. This article will address two questions. The first is whether it is possible in practice for the Court of Protection to identify P's wishes and feelings and having identified their substance to integrate them into its assessment of what is in P's best interests. The second is the ethical question of whether the Court of Protection *should* be concerned with P's wishes and feelings. In order to answer the first question the article analyses a number of recent Court of Protection decisions which demonstrate why engaging with P's wishes and feelings is challenging. The challenges raised by evidence of wishes and feelings turn on whether the claim P is making can be identified, if identified whether it seems plausible and if plausible whether it can be acted upon. This is not to the Court's discredit since all three factors make reaching a convincing best interests decision difficult. What is problematic is that the judgments analysed below do not state that P's evidence is flawed or confusing and the difficulties the judge has in weighing it are therefore not described or resolved. The problem is intensified when P has wishes or feelings which are extremely complicated for P to express. In these circumstances the Court has to both identify whether meaning can be elucidated from P's statements regarding her wishes and feelings, and then establish what value to apply to them. In the case of people who lack capacity, situations where wishes and feelings are not clearly expressed are perhaps more likely to arise than not and these situations therefore deserve careful consideration.

In analysing why the problems raised by analysing P's evidence and the inconsistencies in judicial reasoning that ensure from this are a problem for the Court of Protection this article makes the argument that the status quo undermines the dignity of P as a participant in the decisions being reached about her life. If we accept that the Court's jurisdiction is a necessary corollary of the operation of the Mental Capacity Act and that some form of due process is a necessary adjunct to any state involvement in the lives of people held to lack capacity we further need to ask whether the processes by which we reach decisions upon P's behalf *should* be constitutive of P's dignity? If they are, this imposes ethical obligations upon judges in the Court of Protection to engage more deeply with the substance of any evidence obtained from P concerning her wishes and feelings. This article does not challenge the MCA framework which assumes that P's current wishes and feelings are only one type of evidence to be taken into account when determining what decision is to be reached. But construing dignity as arising through process does demand that reasonable analysis is made of P's evidence, structural barriers to giving effect to P's wishes and feelings are identified as sources of injustice, assumptions made prior to hearing P's evidence are re-evaluated, and the reasons for rejecting P's evidence as implausible or leading to conclusions which are practically unworkable are stated plainly. This is important because it enables P to challenge the decisions which affect her life and keeps P within the community of actors that the Court is communicating with and not merely about.

### Can the Court of Protection identify what P's wishes and feelings are?

Two assumptions are made in S. 4(6)(a) of the MCA. The first is that people have or had wishes and feelings, and the second is that where reasonable efforts are made by others it *may* (not will) be possible to establish what these are.

For many of the people whose lives are affected by the MCA this is obviously true and we should welcome a best interests test that recognises that this is the case. The ‘balance sheet’ approach to evaluating best interests often favoured by the courts before the MCA was adopted did not identify the wishes and feelings of P after she had been identified as lacking capacity as salient (see Thorpe LJ in *Re A (Medical Treatment: Male Sterilisation)* [2000] 1 FCR 193 at p. 206). But even if P lacks the capacity to reach a decision about the merits of taking anti-epilepsy drugs to reduce the risk of seizures, P may still wish not to have seizures, or have feelings about swallowing pills or experiencing side-effects such as depression or weight gain as she adjusts to the drug. The person making the decision on P's behalf should consider those wishes and feelings when deciding whether and how to treat P's epilepsy.

But what about situations where P cannot identify her own wishes and feelings? Or wants multiple outcomes which conflict? Or where P has wishes or feelings which she is fully aware of but wishes to conceal? All of these circumstances arise frequently in daily life. P may be so emotionally distraught that she cannot coherently articulate her current feelings. P may wish to become pregnant and therefore may simultaneously want to control her epileptic seizures with medication but also wish to come off medication to reduce the small risk of teratogenic harm it poses to a developing foetus. P may be seriously ill and feel great pain but deliberately conceal this from her spouse so that he does not worry unnecessarily about her.

If P lacks capacity to make a decision, the person charged with making a decision on her behalf is placed in a difficult position in these circumstances. He or she will know that P has wishes and feelings that deserve attention and should be considered in any decision to be taken but will struggle to do this.

### How do wishes and feelings relate to each other?

The Mental Capacity Act consistently refers to both wishes and feelings, implying that the two terms are conceptually distinct and that both must be given consideration. Clearly wishes and feelings are not synonymous but the two terms are never defined separately. The MCA Code of Practice provides no guidance on how to ascertain P's feelings as opposed to P's wishes. The rulings of the Court of Protection have, as yet, not suggested that anything material turns on the distinction between wishes and feelings.

There are good reasons for thinking that continuing to differentiate the two terms whilst linking them together is the appropriate way forward although it would be helpful if the Code of Practice could be more explicit about the distinction between them. A significant body of research in the philosophy of the emotions (Goldie, 2012) neuroscience and neuropsychology (Damasio, 1998) now argues that wishes and feelings are interdependent: the ability to experience emotions may be critical to the ability to formulate plans for the future and make choices about different outcomes (Charland, 1998) or to make moral choices (Stenner, 2005). Studies in this field have tended to focus on the significance of these findings for capacity assessment and the debate has centred upon whether we need to know the extent of P's emotional awareness before we can reach a formal conclusion about her capacity (Banner, 2012; Charland, 2006; Tan, Stewart, A., Fitzpatrick, R. and Hope, T., 2009; Tan, Stewart, Fitzpatrick and Hope, 2006). If wishes and feelings are taken into account even after a determination that P lacks capacity has been made then the relevance of this debate needs to be re-examined. Perhaps the existence of wishes implies the existence of feelings even if P cannot clearly articulate the relationship between the two and this should place us on inquiry. For example, in the case *of Re H (A Local Authority* v *H* [2012] EWHC 49 (COP)) it was held that a woman with autism lacked capacity to consent to sex even after she had been having sex with multiple partners and made every effort to continue to do so despite being deprived of her liberty to protect her from harm. Hedley J concluded that Miss H lacked the necessary emotional awareness to make appropriate choices about sex, whilst having a high level of knowledge of the subject (Re H at paras 17 and 20). But we could argue that Miss H evidenced through her behaviour her wish to engage in sex and that this implied underlying sexual feelings. Perhaps these would be capable of coherent articulation with appropriate assistance but were currently too obscure for the Court to engage with. Alternatively where P can articulate feelings but cannot crystallise these feelings into a clear expression of wish as to outcome this may provide some indication of P's potential for either recovering capacity or enjoying greater decision-making capacity with support now.

### How does the Court respond when people display clear and consistent wishes and feelings?

The statutory framework requires that when an individual lacking capacity expresses a clear and consistent preference for a specific outcome this has to be considered alongside the other dimensions of the best interests test. This is also the guidance given in the Mental Capacity Act Code of Practice (DH 2005 para. 5.38). Prior to the introduction of the MCA the common law framework for making decisions on behalf of people who lacked capacity emphasised that decisions should be made on the basis of their best interests but no weight was attached to their currently ascertainable wishes and feelings. It was presumed that once the individual lacked capacity their wishes and feelings no longer pertained to the decision to be reached. So we have to refer to the case-law after the MCA was passed to find any direction on how P's current wishes and feelings are to be weighted against other factors in reaching a best interests decision under the Act. This has been unhelpfully vague. The clearest instructions were provided by Munby J in the case of *ITW* v *Z* ([2008] EWHC 3425 (Fam)) who found that: ‘… the statute lays down no hierarchy as between the various factors which have to be borne in mind’ (*ITW* v *Z* at para. 32). When considering S. 4(6)(a) MCA he stated that:

… the weight to be attached to P's wishes and feelings will always be case specific and fact-specific. In some cases, in some situations, they may carry much, even, on occasions, preponderant, weight. In other cases, in other situations … they may carry very little weight. One cannot, as it were, attribute any particular a priori weight or importance to P's wishes and feelings; … And even if one is dealing with a particular individual, the weight to be attached to their wishes and feelings must depend upon the particular context; … Just as the test of incapacity under the 2005 Act is, as under the common law, ‘issue specific’, so in a similar way the weight to be attached to P's wishes and feelings will likewise be issue specific. (*ITW* v *Z* at para. 35)

Despite the lengthy reasoning Munby J's judgment is not informative. P's wishes and feelings will be taken into account to the extent that they are relevant and significant but no more. As with so many other matters to be decided by the Court of Protection the matter is ultimately both ‘issue specific’ and ‘case specific’ but it does not logically follow that because the test of incapacity under the MCA is ‘issue specific’ the weight to be attached to P's wishes and feelings must be also.

At best, Munby J's guidance is not to overstate the value to be placed upon P's wishes and feelings after it has already been determined that P lacks capacity. From that point onwards the Court is enjoined to adopt a broad perspective and exercise its discretion. This does not mean that the court will not place considerable emphasis upon P's wishes and feelings where they can be identified and where they support the judge in reaching her decision. An example of this is given in the case of *Re JH and MH* ([2011] EWHC 2420 (COP)). Mrs H had suffered a stroke and was in hospital waiting to be discharged. The local authority applied for an order from the Court of Protection that it would be in Mrs H's best interests to be discharged to a nursing home. Eldergill J. held that:

Mrs H's views in this case have always been, and are, clear and consistent:

- She wishes to return home and to live with her husband.
- She remains committed to her husband, marriage and home and is devoted to him. Being separated from him and her home would cause her great distress.
- Such a wish is consistent with her values as the partner to a long marriage and must be given very significant weight.
- She wishes her husband to make decisions on her behalf.
- As with any person who is devoted, she wishes to take her husband's feelings and wishes into consideration. She would want to know that he is as happy as possible, and is not distressed by the outcome.
- She prefers to have limited contact with professional carers and has sometimes resisted the delivery of care by professionals. (Re JH and MH at p. 42)

The wider evidence demonstrated that Mrs H had previously been cared for successfully by her husband for many years and given these facts and the evidence as to her wishes the judge had no difficulty in finding that it was in her best interests to return home notwithstanding the difficulties the local authority faced in funding and delivering an appropriate care package for her.

### How does the Court respond when people demand things which are inconsistent with what the Court of Protection can order or that service providers can deliver?

Mrs H's case demonstrates that the Court will make orders which may be difficult for service providers to honour. The local authority in that case had struggled to deliver effective homecare to Mr and Mrs H in the past, and the district nursing team had encountered problems visiting their home. In addition, Mr H's expectations about the care package to be funded were inconsistent with what the local authority felt able to deliver and this had led to a very strained relationship. There are other instances of the Court of Protection making orders which result in local authorities or healthcare providers incurring significant financial liabilities (*KT* v *CC and STCC* [2012] EWHC 2136 (COP)) and also having to resolve very difficult relationships with understandably aggrieved service users (*Neary* v *Hillingdon* [2011] EWHC 1377 (COP)).

This section looks at the bigger problem which arises when P expresses wishes and feelings with great clarity on matters which the Court rules it does not have the legal authority to resolve. In *Re C (C* v *Blackburn with Darwen Borough Council and A Care Home and Blackburn with Darwen Teaching Care Trust* [2011] EWHC 3321 (COP)) Mr C had learning disabilities and epilepsy and was deprived of his liberty in a residential home (subject to the Deprivation of Liberty Safeguards or DOLS found in Schedule A1 Mental Capacity Act 2005 which allow local authorities to authorise detention in social care facilities where an individual lacks the capacity to decide where to reside and the placement is in her best interests). He was also subject to a guardianship order under S. 7 of the Mental Health Act 1983. He sought an order from the Court of Protection that both the local authority's decision to authorise his deprivation of liberty and the guardianship order were unlawful. The evidence as to Mr C's wishes and feelings was straightforward:

Mr C spoke on his own behalf. He said that he feels a great deal of stress as a result of being under both guardianship and DOLS. It gets on his mind and makes his epilepsy worse. He gets confused and doesn't know who to turn to. He has been at the care home for two years and wants to go somewhere else. He does not get on with the other residents and stays in his bedroom for most of the day to avoid them. The situation is too much for him. He wants the guardianship and the DOLS lifted. (Re C at para. 19)

Not only did Mr C state that he found living in his current home bad for his health, but this view was supported by an independent social worker appointed by the Official Solicitor who advised the Court that the current residential home placement was not in Mr C's best interests.

Jackson J. found that Mr C was not subject to a deprivation of liberty because his liberty in the home was simply restricted by the limitations placed upon his movements and the fact that he could not go out unaccompanied. Turning to the point that Mr C wanted to move to another home the judge made the following observation:

In the present case Mr C undoubtedly wants to live somewhere else, but this is a reflection of his unhappiness with the care home. He would like to be able to live an unconfined life in the community, but this is not realistically possible due to the extent of his difficulties. I distinguish his situation from those where a person has been removed from a home that is still realistically available. (Re C at para. 26)

For our purposes what is problematic is the bald assertion by the judge that Mr C ‘would like to live an unconfined life in the community’. This is not supported by Mr C's evidence, nor was it a claim made on Mr C's behalf by the Official Solicitor. The Official Solicitor only requested that the local authority consider the placement recommended by the independent social worker instead of the current residential home where Mr C was unhappy. Mr C might aspire to live independently, and he had lived independently in the past, but his wishes and feelings as stated, and as they pertained to the decision to be taken in this case, seem to be limited to moving on from this particular care home. The judgment may have been coloured by the fact that the judge had already found that he did not have the power to order that the guardianship order which applied to Mr C under the Mental Health Act be lifted. This empowered whoever was acting as guardian (in this case the local authority) to require Mr C reside at a place it directed. For as long as the Mental Health Act Guardianship was in effect any order regarding deprivation of liberty made by the Court of Protection would be redundant. The only way to get the guardianship order lifted would be to apply to the Mental Health Tribunal which Mr C had already done unsuccessfully. Thus regardless of how the judge interpreted Mr C's evidence, or what weight he thought it proper to attach to it in deciding what was in Mr C's best interests it could have no practical effect on the outcome of the case.

### How does the Court respond when people wish for irreconcilable outcomes?

In the cases of *Re JH and MH* and *Re C*, P presents evidence which is easy to interpret even if it is not easy for the Court to translate into a ruling or for service providers to then act upon. The dilemma of how to resolve issues which seem incapable of resolution is far more acute when P provides evidence regarding her wishes and feelings which seems internally contradictory or wholly at odds with anything which could be ordered by the Court. This issue is exemplified by the facts in E v A Local Authority ((Medical treatment: Anorexia) (Rev 1) [2012] EWHC 1639 (COP)). The case concerned E, a 32-year-old woman who had suffered anorexia nervosa since her early teens. For the two years before the case came before the Court of Protection her BMI had been dangerously low she had experienced repeated hospitalisations. In March 2012 E was detained in hospital under S. 3 of the Mental Health Act 1983. Naso-gastric feeding was started although E refused all treatment. At a meeting between E, her family and care-team it was agreed that all potential treatment options had been exhausted and a palliative care pathway was introduced. Naso-gastric feeding was stopped and E was transferred to a community hospital. She was also discharged from S. 3 detention.

Some six weeks later an urgent application was made by E's local authority to the Court of Protection regarding this decision to withdraw life-sustaining treatment from an adult who lacked capacity. Its concern was that E's status as an individual protected by the Mental Capacity Act had been overlooked.

The judge, Jackson J., found that E did not have the necessary capacity in April 2012 and that she had not created a valid advance refusal of treatment when she had capacity. That being so he then had to make a decision regarding what treatment would be in her best interests.

The options he identified were to continue with the palliative care pathway already agreed upon in which case E's death would be inevitable, or to transfer her to an intensive care unit within a specialist hospital and reinstate refeeding, forcibly (i.e. under sedation) if necessary, until such time as E's BMI was higher and the subsequent improvements in her physical health enabled her to engage with psychotherapy. The prognosis was uncertain. E's physical frailty was so great that her chances of surviving or not surviving treatment were described as equally balanced. Should she survive, two psychiatrists who specialised in the treatment of eating disorders giving evidence to the Court assessed the likelihood of recovery ‘in the sense of returning to a quality of life that she [E] would regard as being worthwhile’ at around 20% (Re E at para. 72).

After evaluating the evidence of the medical experts and E's parents, and considering the conflicting values of E's personal autonomy and the protection of her life the judge ordered that she be transferred to a specialist hospital where she could be detained under the MHA again and compulsory treatment could resume.

A representative of the Official Solicitor was able to see E in hospital to obtain a statement regarding her current wishes and feelings. This statement is worth quoting in full:

She [E] recognises that everything that could have been done to help has been tried she has endured a lot of pain with very little benefit. She describes her life as ‘a pure torment’. All the things she had wanted to do have proved impossible because of her illness. She can't achieve any of her goals and she feels crushed. She feels she is in a situation where she is able to give nothing to the world and the world is able to give nothing to her. She is conscious that she represented a huge burden to her family, who have always been extremely supportive and caring. She feels that her life is pointless. She would have wanted to have a relationship with her sisters’ children but because of her condition that is not possible. She has tried to explore every avenue to get over her demons but has failed. She wants to live for the remainder of her life as she chooses, and if necessary to be allowed die with dignity. She understands that she will die without intervention. She does not want to be killed, she just wants to be allowed to act as she wants. It is possible that she might change her mind, but not because somebody forces [her] to do so. She has core beliefs and it will be those beliefs that will determine if she wants to stay alive. She asks the court to respect her wishes. (Re E at para. 76)

The judge states without qualification that E wishes for a treatment plan that would lead inevitably to her death (Re E at para. 115). He made no efforts to engage with the subtleties of E's views, and although one medical expert suggested that E's views could be characterised as ambivalent (para. 99) the judge did not address this point in his reasoning.

The judgment attracted considerable media attention when it was published, perhaps regrettably since E had done nothing to encourage this. It was characterised as ‘a right to die’ case and divided opinion along predictable lines. Some media commentators viewed it as an example of the Court overstepping its authority and failing to heed the wishes of E or her parents (Hewson, 2012; *Observer*, 2012). Others saw it as a desirable statement by the Court that it will take proactive steps to protect life in extreme cases (Marsden & Beckford, 2012). But closer scrutiny of E's evidence suggests her wishes primarily concerned her right to physical integrity rather than her right to die.

E's evidence indicates that she had a very nuanced and subtle awareness of her situation, albeit one which could not lead to a simple resolution. E felt hopeless about the future: ‘… everything that could have been done to help has been tried’, ‘her life is pointless’, ‘she has tried to explore every avenue to get over her demons but has failed’. She also felt: ‘a lot of pain’ and her ‘life is a pure torment’. Finally she felt thwarted by her illness and the impact it had upon her life: ‘She can't achieve any of her goals’, ‘She would have wanted to have a relationship with her sister's children but because of her condition that is not possible’. But E makes much more equivocal statements regarding her wishes as to the future. She did not say that she would like to die. She wanted to ‘live for the remainder of her life as she chooses’ and ‘does not want to be killed’ and only ‘if necessary [does she wish] to be allowed to die with dignity’. In fact she cautiously indicated that she might choose to stay alive, but that this would not be ‘because somebody forces her to do so’. She clearly wished to retain control over her body.

The difficulty with the judgment was that as presented there were only two viable treatment options: one representing certain death and the other a very small hope of life. But E's evidence complicated matters. E felt that her current situation was untenable, that exerting control over and forcing her to live was also unacceptable, but that was not the same as expressing a wish that she would positively desire to die. E referred in her statement to a third possibility; that left alone she might decide to change her mind. But in the judgment it was stated this was so unlikely to occur as to be ‘negligible’ before E's evidence was even quoted (Re E at para. 45). The judge's appraisal might well have been right. Certainly the medical experts consulted on E's case agreed with him and the wider evidence on the prognosis for eating disorders as severe as E's would tend to support his views. Nevertheless, E no longer wished to be ‘interfered with’. This was also expert evidence; it required precise analysis and evaluation too.

In this instance closer analysis of E's evidence would have allowed for a reordering of the judgment that might have silenced *some* of the criticism raised after the case was heard. The judgment as it stands decides that although E desires to die, since she lacks capacity and it could not be in her best interests to die, even though intervention will be arduous, distressing and possibly futile, it is still better than the alternative. But it is arguable that E did not desire to die, she desired to live, albeit without anyone interfering with her body. Those terms were not such that they gave any viable prospect of her actually surviving, but since she showed some underlying interest in living longer the Court could argue that it was justified in ordering that alternative and more invasive treatment be given instead.

### How does the Court respond when people want to conceal their actual wishes and feelings?

The final set of problematic cases we can look at concern situations where P's ‘true’ wishes and feelings are assumed to be obscured either by the undue influence of another, or by P's generous desire to protect the feelings of her loved ones. These cases arise fairly frequently. One example is the case of *Re A and A* (*A Local Authority* v *Mrs A and Mr A* [2010] EWHC 1549 (Fam)). Mrs and Mr A had learning disabilities and had been married for 18 months when their local authority applied to the Court of Protection for an order making declarations that she lacked capacity to make decisions relating to contraception and that it would be in her best interests to use contraception.

Mrs A had two children prior to meeting Mr A. During each pregnancy she had been assessed as unable to raise a child independently and both children had been removed from her care and placed for adoption at birth.

Mrs A's evidence was complex. Some of it concerned her wishes regarding her relationship with her husband and her plans to have children, some of it concerned her feelings regarding the use of long-term contraception, some of it concerned her feelings regarding the way her husband treated her. Unlike the evidence in the cases discussed above where the statements made by P were either delivered in Court or made to a representative of the Official Solicitor, the judge considered evidence regarding Mrs A's wishes and feelings from a number of other witnesses too, most of them employees of the applicant local authority.

In the event the judge, Bodey J, found that Mrs A did not have capacity to make decisions regarding the use of contraception. He also evaluated whether to make an order regarding what would be in Mrs A's best interests. Here he found that any attempt to compel Mrs A to use contraception would require the use of force and would be practically impossible. He was not prepared to make an order to this effect.

In this case it is not the judge's assessment of what is in Mrs A's best interests that is problematic. Instead, it is how he reaches his assessment that Mrs A lacks capacity based upon her wishes and feelings which seems unwieldy. He considered arguments from representatives of the local authority that capacity to use contraception required the capacity to understand the implications of having and raising a child but discounted this line of argument, favouring a more minimal test. To make a decision regarding contraception it was sufficient for Mrs A to understand:

the reason for contraception and what it does (which includes the likelihood of pregnancy if it is not in use during sexual intercourse);the types available and how each is used; the advantages and disadvantages of each type; the possible side-effects of each and how they can be dealt with; how easily each type can be changed; and the generally accepted effectiveness of each. (Re A and A at para. 64)

Mrs A demonstrated this level of knowledge. However, he then considered whether she had the ability to ‘use or weigh’ this information in reaching her decision as required by S. 3(1)(c) MCA and concluded that she did not due to ‘the completely unequal dynamic in the relationship between Mr and Mrs A’ (Re A and A at para. 73). Mr A exerted undue influence over her behaviour. This was demonstrated by a consistent pattern of bullying and violence towards her. The judge ignored one significant explanation Mrs A provided for her refusal to use the long-term depot injection as a form of contraception, which would otherwise be suitable for her since her husband was not willing to take responsibility for contraception himself and discouraged her from taking daily medication. Having previously used the depot injection for more than six months Mrs A stated at least three times that she did not want to use it any longer because it made her stomach distended and gave her backache. These are fairly common side-effects of progrestogenonly forms of contraception. She said this to her college course coordinator (Re A and A at para. 18), to the representative of the Official Solicitor (para. 37) and to one of the medical witnesses (para. 49). She also informed the representative of the Official Solicitor that she wanted to experience her periods (para. 37). Again amenorrhoea is a side effect affecting the majority of women using the depot contraceptive injection. At no point does the judgment consider that her refusal of contraception might be influenced by the effects it had upon her body. Instead, the evidence that she had been concealing her feelings was deemed so overwhelming that the judge privileged evidence which revealed what he presumed to be her ‘true’ feelings:

Miss S's [Mrs A's college course coordinator] evidence is compelling that in the early part of 2009 Mrs A was scared when she thought she was pregnant and was asking if there was any contraception which she could take without Mr A knowing [This] is significant because it was not in answer to formal questioning, but was a spontaneous remark, woman to woman. It showed clearly Mrs A's true wishes and feelings at that time. (Re A and A at para. 69)

Given that she indicated her knowledge of contraception and cogent reasons for refusing contraception, the conclusion that Mrs A's refusal was attributable primarily to her husband's undue influence is problematic. Mrs A might have objected to depot injection contraception because it had unpleasant side effects for her and she had been married to a man who wished her not to use contraception for unrelated reasons. That is not to conclude that his undue influence was not of greater significance in determining her decision to use contraception than her independent feelings regarding side-effects. Evaluations concerning undue influence will always be a matter of degree, and in any judgment of this nature there will always be the theoretical possibility that P would, independent of influence, have reached the same decision as the one she has made under the influence of others. Butler-Sloss LJ indicated how hard the influence maybe to disentangle in *Re T*: ‘… both at law and in equity it has long been recognised that an influence may be subtle, insidious, pervasive’ (*Re T.* [1992] EWCA Civ 18 at para. 50). Notwithstanding the finding of lack of capacity the judge invoked the inherent jurisdiction of the Court to safeguard vulnerable adults to encourage Mr A to enable Mrs A to have unfettered contact with professionals, and to reduce the degree of coercion he exercised over her. The High Court even after the passing of the Mental Capacity Act had decided in the judgment in *Re SA* ((Vulnerable Adult with Capacity: Marriage) [2006] 1 FLR 867) that it retained jurisdiction to make safeguarding decisions with regard to adults who might otherwise be at risk of significant harm. This rather piecemeal approach to developing a safeguarding jurisdiction in England and Wales has been criticised because it lacks the coherence of a statutory jurisdiction, and relies on a poorly defined concept of vulnerability which has been developed by the courts rather than by parliament (Dunn, Clare & Holland, 2008; Keywood 2011). Since evidence concerning undue influence is often complex and the Court's jurisdiction in this field is controversial it seems more critical rather than less that the characteristics of Mrs A's wishes and feelings be explored fully in order to render the judgment plausible. Mrs A's learning disability and Mr A's coercion both needed to affect her decision regarding contraception in order for the Court to be able to exercise its jurisdiction in this case since the jurisdiction of the Mental Capacity Act did not apply (*A Local Authority and others* v *DL* [2012] EWCA Civ 253 at para. 78). On the basis of the facts as described it is not clear how Mrs A's learning disability made her specifically vulnerable to Mr A's coercion and there is even some evidence that she had independent reasons for making a decision which the local authority would prefer her not to.

### The legal justification for taking P's wishes and feelings more seriously when reaching decisions in the Court of Protection

The cases described here represent the tip of an iceberg. Only a minority of decisions made by the Court are reported since Rule 91 of the Court of Protection Rules 2007 gives the Court a wide ranging discretion to control the publication of their decisions in order to protect the privacy of the parties. In those cases that are reported only a minority contain express evidence from P concerning her wishes and feelings. So the examples described are unlikely to be representative of the bulk of the Court's day-to-day work, however they are likely to be unrepresentative in a manner which gives the Court the opportunity to show its best rather than its worst practice since all involve hearings where evidence from P is at least considered.

Even where an individual's current wishes and feelings have been clearly and consistently expressed, knowing how to reach a decision which takes these into account alongside the other sources of evidence regarding P's interests outlined in s. 4 MCA is not straightforward. The drafters of the Code of Practice clearly concluded that a rigid formulation would be unhelpful for frontline decision-makers. The judges in the Court of Protection reached the same conclusion which is why case-law has emphasised that each of these factors needs to be treated as both case-specific and issue-specific. However, as the discussion above demonstrates, there are a number of reasons why the failure of the Court to give consistent regard to P's evidence concerning her wishes and feelings makes it easier to criticise their judgments, both for being flawed procedurally and failing to give due regard to all relevant evidence, and in consequence for being flawed as to outcome.

There are also a number of reasons why this is legally problematic. The first is that the Court is not meeting its own statutory obligations. S. 4(6)(a) MCA enjoins us to have regard to P's past and present wishes and feelings when reaching a decision on her behalf, and in some cases they simply are not. The Court of Protection Rule 3.1 states that their overriding objective is to ensure that the Court deals with cases justly having regard to the principles in the MCA. The Court is at best not developing a coherent line of jurisprudence and at worst failing to follow earlier decisions. Of course it is not bound to do so, but it would be extremely helpful if it did.

Beyond the domestic legal situation, the practice of the Court is out of step with developments in the Strasbourg jurisprudence. The judgment in *Shtukaturov* v *Russia* ((2008) MHLR 238, (2012) 54 EHRR 27) found that representation of evidence concerning a person's wishes by an ‘interested party’ such as a hospital representative at a hearing did not amount to adequate protection and that, at a minimum, visual contact and, ideally, questioning should take place between a person allegedly lacking capacity and the judge trying her case. The absence of independent evidence concerning the interests of the person allegedly lacking capacity in that case contributed to a violation of the right to a fair trial under Art. 6(1) ECHR. Further to this Art. 12(2) of the UN Convention on the Rights of Persons with Disabilities (CRPD) requires that state parties to the Convention ‘recognize that persons with disabilities enjoy legal capacity on an equal basis with others in all aspects of life’ and Art. 12(3) requires that they ‘take appropriate measures to provide access by persons with disabilities to the support they may require in exercising their legal capacity’. The UK has signed and ratified the CRPD. The starting point for Art. 12 CRPD is a presumption of capacity that cannot be rebutted by reference to any related disability status since this would, in itself, be discriminatory. Bartlett (2012, pp. 762–763) points out that the structure of the MCA is fundamentally at odds with this because a framework which relies on a finding of ‘an impairment of, or a disturbance in the functioning of, the mind or brain’ (S. (2) MCA) before introducing a substituted decision-making framework cannot actually be reconciled with Art. 12. Nevertheless since Art. 12 requires state parties to make all necessary incremental steps towards realising the goal of equal legal capacity, ensuring that the evidence of people whose mental capacity has been called in to question is given greater weight when reaching decisions about their lives might be a significant first step in realising this goal. It is therefore unfortunate that the UK Government is currently ignoring this issue (Office for Disability Issues, 2011).

### The problem with autonomy as a normative basis for justifying taking wishes and feelings seriously

Given the legal and pragmatic justifications for taking P's wishes and feelings seriously it should be easy to make out the ethical argument that the Court must give this evidence serious consideration and explain its use of the evidence fully in its judgment. Indeed it can be argued that it is self-evident that P's evidence should be treated as of primary significance (Minkowitz, 2012). But arguments from self-evidence are awkward and careful scrutiny suggests the ethical arguments for affording P's evidence closer attention cannot be presumed to rely on a single norm.

The Mental Capacity Act Code of Practice (Department for Constitutional Affairs, 2007) states that the presumption in law that adults retain the capacity to make decisions upon their own behalf in almost all circumstances, even where those decisions may appear unwise can be characterised as a ‘right to autonomy’ (para. 1.2). This value has also been espoused in the case-law of the Court of Protection although the Court recognises that personal autonomy may be just one dimension of the overall picture of best interests for an adult lacking capacity. Other interests which the Court has tried to uphold include the protection of the vulnerable adult from harm (see *Re F* [2009] EWHC B30 (COP)) and the preservation of happy memories after death (Re P [2009] EWHC 163 (COP) at para. 44).

We could argue that the logic of S. 4(6)(a) MCA regarding the need to take P's wishes and feelings into account in reaching a decision concerning her best interests is straightforward: Evidence concerning P's wishes and feelings deserves to be given consideration, irrespective of whether it indicates a wise decision, because it is the best available evidence of P's surviving autonomous desires. The law takes autonomy seriously and defends the right of P to make unwise decisions when P retains capacity. The function of S. 4(6)(a) MCA is to ensure that even where we have held that P does not have capacity to make the decision all residual preferences are respected. The Court of Protection should make every effort to address this evidence in its reasoning in order to communicate that justice is not only being done but being seen to be done.

But there are two reasons why this is not satisfactory. The first relates to the operation of the Court itself and the second relates to how we define autonomy.

P does not usually choose to go to the Court of Protection. We can assume that it is necessary in a compassionate society to enable others to make decisions upon P's behalf, where P lacks the capacity to make meaningful decisions for herself. We can assume that it follows that these decisions should be transparent, open to challenge where necessary and subject to due process of the law where required. But it does not follow from either of these premises that P must choose to share evidence about her wishes and feelings. The Court of Protection has made specific rulings on whether individuals have the capacity to consent to marry, engage in oral, vaginal or anal intercourse or sexual touching, use contraception or refuse live-saving treatment. In none of these cases is it necessary or even especially likely that P would wish to share her wishes and feelings concerning the matter with the Court. It is paradoxical to argue that the Court has the moral authority to enquire into a person's feelings regarding sexual intimacy purely on the basis that it must to do so to protect that individual's autonomy.

Privacy is, of course, a contested concept. But this problem persists regardless of how we construct P's underlying interest in her privacy. Whether privacy is construed as freedom of choice (Feldman, 1994), the ability to exercise control over one's social identity (Michael, 1994) or the ability to enjoy secrecy and solitude (Gavison, 1980) the problem remains: the practice of gathering evidence for the Court of Protection hearing violates P's privacy. In this context it does not matter that P may not actively object to the evidence gathering process since we have already determined that in most cases P will lack the capacity to litigate and may therefore also lack capacity to raise complaints concerning her treatment.

The second problem of what meaning attaches to autonomy in this context has already been extensively explored in the critical literature on the operation of the Mental Capacity Act (Chico & Hagger, 2011; Coggon & Miola, 2011; Owen, Freyenhagen, Richardson & Hotopf, 2009). In its simplest terms there is a tension between the Kantian conception of autonomy in which the ability to exercise autonomy is directly related to rationality (and inversely related to loss of mental capacity) and a different notion of autonomy as the ability to exercise self-determination more commonly employed in legal decision-making. Whilst philosophers have worked hard to expand and refine their conceptions of autonomy to accommodate these complexities (Freyenhagen & O'Shea, 2013; Radoilska, 2012) the legal system has not responded accordingly. This is particularly evident when we look at the use of autonomy in the law on mental capacity where post-MCA the value of autonomy is often employed after the fact that P lacks capacity to reach a decision has been established. At this point it has already been determined that P has a disorder affecting her mental capacity and may have reduced capacity to formulate meaningful wishes, reduced capacity to express these wishes and reduced awareness of the interests of others and reduced responsibilities towards others as well. In many relevant senses her rationality and capacity for self-determination are profoundly compromised by these circumstances. The Court is aware of the ambiguity of the concept/s of autonomy they employ and have consistently reserved the right to decide future cases on a different basis with regard to different values. Using autonomy as a norm therefore serves as a poor indication of future decision-making by the Court, which is one of the key functions of identifying legal norms. And it only minimally protects P's interests since the Court does not employ a coherently defined model of autonomy based around a fully developed conception of the self.

### Taking wishes and feelings seriously: the protection of P's dignity

Nevertheless, the statute requires P's wishes and feelings be taken into account even after it has been determined that she can no longer make a decision on her own behalf. And it seems intuitively obvious that this is a morally important thing to do. So what value is being protected by this decision-making process? I have argued above that autonomy is too thorny a concept, here I wish to argue that human dignity serves as a better guiding value to define what the Court of Protection should be aiming to achieve in its decision-making processes.

It has been observed in recent writings on dignity that the term functions as a linguistic placeholder enabling a wide range of disparate and sometimes conflicting meanings to become a focal point for communication (Habermas, 2010; McCrudden, 2008 at pp. 679–680). In a wide-ranging review of the uses of the concept of human dignity in judicial interpretation McCrudden (2008) describes a ‘minimum core’ which seems consistent within and between the differing jurisdictions which employ it. This comprises three components:

The first is that every human being possesses an intrinsic worth, merely by being human. The second is that this intrinsic worth should be recognized and respected by others, and [thirdly] some forms of treatment by others are inconsistent with, or required by, respect for this intrinsic worth. (McCrudden 2008, p. 679)

McCrudden goes on to point out that in practice human dignity is used to justify positions in different jurisdictions which often strongly contrast with each other and occasionally imply irreconcilable differences in the underlying conceptions of what it means to enjoy dignity. These differences are rarely crude and frequently arise from a complex interplay between constitutional documents and the roles played by constitutional courts and parliaments. This indeterminacy undermines human dignity's usefulness as a global value around which we can build a substantive consensus beyond the minimum core. McCrudden's minimum core is merely descriptive of how dignity functions. It lacks a normative dimension which could indicate how people who lack capacity should be treated when decisions concerning their lives are to be made by the courts.

In the Western tradition, dignity is also problematic for advocates on behalf of those lacking capacity because Western proponents of dignity often link the concept to the Kantian concept of autonomy. A good example is provided by Waldron:

[human] Dignity is the status of a person predicated on the fact that she is recognised as having the ability to control and regulate her actions in accordance with her own apprehension of norms and reasons that apply to her; it assumes she is capable of giving and entitled to give an account of herself (and of the way in which she is regulating her actions and organising her life), an account that others are to pay attention to; and it means finally that she has the wherewithal to demand that her agency and her presence among us as a human being be taken seriously and accommodated in the lives of others, in others’ attitudes and actions towards her, and in social life generally. (Waldron, 2012, p. 202)

Waldron's conception of human dignity is predicated on the assumption that people as bearers of the status of dignity are capable of apprehending norms and bringing them to bear on their own actions. Whilst this is clearly true for many people who lack capacity in many areas, it is untrue sufficiently frequently for Waldron's conception to be immediately problematic.

So dignity for our purposes requires a definition that falls somewhere between McCrudden's ‘empty shell’ of dignity in which animals and human foetuses may enjoy some degree of protection as a result of their intrinsic worth despite the fact that they can bear no responsibilities, and Waldron's definition in which dignity is enjoyed on the basis of one's ability to self-govern. Such a definition may be difficult to formulate as a precise proposition, but we have at least identified the continuum it lies upon.

Waldron describes his definition of human dignity as defined by status and not values. Interestingly, in the same article he goes on to describe how law protects human dignity in a number of contexts in which this description of human dignity could, arguably, not be said to apply. Most importantly Waldron argues that law protects human dignity through procedure. A court or tribunal he argues must have the following characteristics:

each party has the opportunity to present arguments and submissions … and answer those of the other party. In the course of all of this, both sides are treated respectfully, and above all listened to by a tribunal which is bound in some manner to attend to the evidence presented and respond to the submissions that are made in the reasons it eventually gives for its decision. (Waldron, 2008, p. 210)

He goes on to justify this by stating that:

… law is a mode of governing people that acknowledges that they have a view or perspective of their own to present on the application of the norm to their conduct and situation. Applying a norm to a human individual is not like deciding what to do about a rabid animal or a dilapidated house. It involves paying attention to a point of view and respecting the personality of the entity one is dealing with. As such it embodies a crucial dignitarian idea – respecting the dignity of those to whom the norms are applied as beings capable of explaining themselves. (Waldron, 2008, p. 210)

This idea of dignity as a norm which applies to process rather than outcome is comparatively underexplored. Most of the literature critiquing and advocating the legal uses of dignity have emphasised, as McCrudden does, the commonalities and disjunctures within and between dignity in determining the outcome of a specific judgment. This is curious because dignity is clearly a concept which historically functioned to determine current conduct rather than the rights we carried forward into the future. As Arendt (1958, pp. 204–205) points out, dignity for the Greeks was attained through labour and action, and had a transient, dynamic, even fragile, character. It is also useful here, because on the face of it, none of the judgments described above necessarily violated P's dignity in their ultimate conclusions, even if the process by which the judgment was reached was undignified. In *Re E*, the decision was so finely balanced that even a careful reappraisal does not automatically lead to the conclusion that painful, prolonged and invasive forced treatment would be an intervention that failed to respect her intrinsic self-worth. In *Re A and A*, the judge did recognise that forced contraception would not be in Mrs A's best interests because it would be so traumatic notwithstanding Mrs A's lack of capacity, Mr A's undue influence and the risks associated with her having another child. It is the neglect of Miss E and Mrs A's perspectives in reaching these decisions which makes us call these judgments into question rather than the outcome that was actually reached.

If we abandon Waldron's problematic definition and just focus on his argument that law protects dignity through procedure we can get closer to defining what the Court of Protection should do with the evidence it receives regarding P's wishes and feelings. As a tribunal it is bound as Waldron suggests ‘in some manner to attend to evidence presented and respond to the submissions that are made in the reasons it eventually gives for its decisions.’ But the reasons for doing so cannot be found in P's status and the law's obligation to protect it, but in the integrity of the process itself. Paying closer attention to P's wishes and feelings in reasoning about what will be in her best interests, even where doing so is difficult, and even where the result may be an express statement that P's evidence is implausible or impenetrable will not automatically protect P's dignity in any of the senses described by Waldron. It will neither render her an actor capable of perceiving the norms which apply to her own actions, nor will it necessarily make others perceive her in this way. Indeed in some of the cases described above, such as *Re E*, had P's evidence been given closer scrutiny by the Court quite the opposite effect might have been achieved.

But the wider effect of ensuring evidence is taken from P and looking for ways to first evaluate and then, if necessary, discount it would be to dignify the Court's processes as a whole. Keeping P within the community of actors the Court is communicating with even where that presents practical problems for the Court ensures that the transient and fragile process of protecting dignity can be sustained through dialogue. It reflects a view that P's ideas about her life retain intrinsic worth which is still not always shared by wider society. Dignity is a useful value to guide decision-making during judicial processes and when reasoning about *how* to reach a judgment because unlike autonomy, even in the absence of a unified definition, we can generally identify it in practice. We can agree that to ask Mr C to give evidence in person regarding his feelings about his home and then to misrepresent those feelings in the process of reaching a judgment is not respectful of Mr C's dignity, regardless of the wider implications of the decision to be reached.

### Conclusion: what should the Court do differently?

Identifying the substance of wishes and feelings and balancing them with the interests of others and with what is practically possible is the stuff of all our most complex interpersonal relationships. The Court of Protection has been charged with the task of delivering sensitive and nuanced decisions upon an infinitely wide range of circumstances and requires discretion to be able to achieve this end. Nevertheless, it is a considerable criticism of its processes rather than the outcomes of its decisions that it has failed to develop a coherent approach to evaluating evidence provided by people lacking capacity concerning their wishes and feelings and in some notable cases has ignored key evidence entirely.

The inclusion of people whose mental capacity has been called into question in the decisions being reached about their lives can be justified on a variety of grounds. It can promote their social inclusion, lead to greater efficiency in making decisions about the allocation of social welfare, reflects a view widely reflected in human rights documents that all actors are free and equal in status regardless of disability and is a fundamental corollary to enjoying various political freedoms (for a summary of these arguments see Lewis & Munro, 2012). But this article which has looked narrowly at the decisions made by one court within one statutory jurisdiction concerning adults lacking capacity has argued that there are two specific reasons why judges should be sensitive to the evidence available concerning the wishes and feelings of people lacking capacity regarding the decisions made about their lives. Ordering judgments so that this evidence is evaluated first, and if necessary reasons for disregarding it are explicitly stated may actually improve the quality of judicial reasoning. And the normative justification for doing this lies in the fact that courts and tribunals which take seriously their obligations to hear the voices of all participants can play a key role in promoting the human dignity of those who come before the law.
